# Current News

**Published:** 1890-08

**Authors:** 


### Current News.

UNION DENTAL CONVENTION, Department of Exhibits, October 28–31, 1890, in Berkeley Hall, corner Berkeley and Tremont Streets, Boston, Massachusetts.—The following fourteen societies will hold a union meeting: Maine, New Hampshire, Vermont, Massachusetts, Rhode Island, and Connecticut State Societies; American Academy of Dental Science, Connecticut Valley Dental Society, Harvard Odontological Society, Harvard Dental Alumni Association, Boston Dental College Alumni Association, Boston Society of Dental Improvement, Worcester Dental Society, New England Dental Society.

You will observe by the above list that we are to have the largest and most representative meeting of the dental profession ever held in this section of the country.

All persons having articles, instruments, and materials of use in dentistry are cordially invited to exhibit the same. Many have already signified their intention of making a large exhibit, and if you desire space, please notify the Secretary at once, giving amount of space you can occupy well, whether floor or table, and height of same. All applications should be made before September 15.

Programmes, with list of exhibitors, issued October 1.

WM. P. COOKE, D.M.D.,
W. E. PAGE, D.M.D.,
H. S. DRAPER, D.D.S.,
*Exhibit Committee.*

Applications to be made to either of the Committee, or addressed to the Secretary,

WM. P. COOKE, D.M.D., *Secretary,*
100 Boylston Street., Boston, Mass.

### OBITUARY.

WILLIAM ROBERT WOOD, L.D.S., aged forty-eight, died at Brighton, England, May 1, 1890. He was widely known in England; was colonel in the volunteers (artillery), and took great interest in many local institutions. He filled high positions in Masonic lodges.

H.

While accepting the "germ theory," Dr. John H. Coyle calls attention to that condition of the secretions of the oral cavity which through action and reaction acts chemically on the dentine, dissolving out a portion of its lime-salts, producing a local habitat for the specific microbes which produce caries, a condition of which many seem to fail to appreciate the real pathological significance.

---

Dr. Wardlaw says it is begging the question to tell patients that the teeth decay because of "a fashionable artificial mode of life," because of "environments," because of "eating slops and soft-boiled food," because of "the modern degeneracy of the human race," etc. These are only predisposing causes, with perhaps a modicum of truth.

---

Dr. Catching suggests covering the top of your operating-table with heavy white paper, over which lay a good plate of glass, with a raised moulding around the edge. This is easily kept bright and clean, and your points are plainly visible.

---

Dr. Jno. H. Coyle says that treatment of alveolar abscess often fails because of necrosis of the apex of the root. The only cure is found in extraction, cutting off the necrosed portion, and replanting.

---

Dr. S. A. White, in regulating, uses the same shaped plate for every kind of irregularity, a vulcanite plate having a band running outside of the incisors and covering the bicuspids and molars, and also a band on the palatine surface.

---

Dr. Catching finds the small porcelain dishes used by photographers and painters for mixing colors very useful about the operating-table; "individual" salts and butter-plates are also handy.

---

Dr. Sid Holland fills roots with gold rolled so thin on a fine broach as scarcely to increase the size of the broach.

